# Territorial black-capped chickadee males respond faster to high- than to low-frequency songs in experimentally elevated noise conditions

**DOI:** 10.7717/peerj.3257

**Published:** 2017-04-27

**Authors:** Stefanie E. LaZerte, Hans Slabbekoorn, Ken A. Otter

**Affiliations:** 1 Natural Resources and Environmental Studies, University of Northern British Columbia, Prince George, British Columbia, Canada; 2 Behavioural Biology, Institute of Biology, Leiden University, Leiden, Netherlands

**Keywords:** Black-capped chickadees, *Poecile atricapillus*, Anthropogenic noise, Masking, Song frequency, Playback experiment, Experimental noise, Vocal adjustment, Urbanization

## Abstract

Low-frequency urban noise can interfere with avian communication through masking. Some species are able to shift the frequency of their vocalizations upwards in noisy conditions, which may reduce the effects of masking. However, results from playback studies investigating whether or not such vocal changes improve audibility in noisy conditions are not clear; the responses of free-ranging individuals to shifted signals are potentially confounded by functional trade-offs between masking-related audibility and frequency-dependent signal quality. Black-capped chickadees (*Poecile atricapillus*) naturally sing their songs at several different frequencies as they pitch-shift to match conspecifics during song-matching contests. They are also known to switch to higher song frequencies in response to experimental noise exposure. Each male produces both high- and low-frequency songs and absolute frequency is not a signal of aggression or dominance, making this an interesting species in which to test whether higher-frequency songs are more audible than lower-frequency songs in noisy conditions. We conducted playback studies across southern and central British Columbia, Canada, using paired song stimuli (high- vs low-frequency songs, *n* = 24 pairs) embedded in synthetic background noise created to match typical urban sound profiles. Over the course of each playback, the signal-to-noise ratio of the song stimuli was gradually increased by raising the amplitude of the song stimuli while maintaining background noise at a constant amplitude. We evaluated variation in how quickly and aggressively territorial males reacted to each of the paired stimuli. We found that males responded more quickly to playbacks of high- than low-frequency songs when high-frequency songs were presented first, but not when low-frequency songs were first. This difference may be explained by high-frequency songs being more audible combined with a carry-over effect resulting in slower responses to the second stimulus due to habituation. We observed no difference in overall aggression between stimuli. These results suggest that high-frequency songs may be more audible under noisy conditions.

## Introduction

Urban noise pollution is generally low in frequency and can interfere with avian communication through masking of overlapping frequencies (Rabin & Greene, 2002; Brumm & Slabbekoorn, 2005; Barber, Crooks & Fristrup, 2010). The greater the extent of frequency overlap, the worse the interference from environmental noise (Lohr, Wright & Dooling, 2003; Pohl et al., 2009) and several studies have found correlations between species persistence in noisy areas and vocal frequency (e.g, Hu & Cardoso, 2009; Proppe, Sturdy & St. Clair, 2013; Francis, 2015; but see Moiron et al., 2015). Theoretical and laboratory-based studies show that vocalizing at higher frequencies should improve detection and discrimination in noise (Nemeth & Brumm, 2010; Pohl et al., 2012). Further, there are many examples of birds singing higher frequencies in noisy conditions (e.g., Slabbekoorn & Peet, 2003; Wood & Yezerinac, 2006; Verzijden et al., 2010; Bermúdez-Cuamatzin et al., 2011; LaZerte, Slabbekoorn & Otter, 2016; LaZerte, Otter & Slabbekoorn, 2017). These lines of evidence suggest that spectral plasticity in response to elevated noise levels may be adaptive, but from field studies there is little direct evidence that spectrally-adjusted songs improve detection and discrimination over unadjusted songs (e.g., Luther & Magnotti, 2014; but see Halfwerk et al., 2011).

Playback studies help determine whether males can differentiate among different stimuli (often song types). These types of studies have shown that the presence of background noise results in slower responses to territorial intrusions in spotted towhees (*Pipilo maculatus*) and chipping sparrows (*Spizella passerina*) (Kleist et al., 2016). Furthermore, playback studies have shown that European robins (*Erithacus rubecula*) adjust their vocalizations in ways that minimize masking—increasing minimum frequency and decreasing song complexity—when responding to territorial intrusions under conditions of elevated background noise (McMullen, Schmidt & Kunc, 2014). However, playback studies addressing whether adjusted songs are both more detectable and discriminable than non-adjusted songs are inconclusive.

Playback studies on great tits (*Parus major*), European blackbirds (*Turdus merula*), northern cardinals (*Cardinalis cardinalis*) and white-crowned sparrows (*Zonotrichia leucophrys nuttalli*) have shown that birds can differentiate between spectrally-adjusted and non-adjusted songs (Mockford & Marshall, 2009; Ripmeester, Mulder & Slabbekoorn, 2010; Luther & Magnotti, 2014; Luther, Phillips & Derryberry, 2016). However, in these studies, spectrally-adjusted songs did not result in a better signal than non-adjusted songs in noisy conditions; great tits and European blackbirds responded most strongly to their local song variants (irrespective of whether it was spectrally-adjusted or not) (Mockford & Marshall, 2009; Ripmeester, Mulder & Slabbekoorn, 2010), and northern cardinals and white-crowned sparrows responded more strongly to non-adjusted (normal) songs than to spectrally-adjusted songs (either frequency adjusted or bandwidth adjusted, respectively) (Luther & Magnotti, 2014; Luther, Phillips & Derryberry, 2016). It seems likely that when spectrally-adjusted songs are atypical to a population, other factors affecting response strength may come into play. Furthermore, the role of masking noise on differentiation is ambiguous; in these studies noise conditions were not experimentally manipulated: they were the local ambient noise conditions of the habitat of the focal male tested. In a study that experimentally manipulated ambient noise levels (examining female responses to male song in great tits) higher-frequency songs were not affected by masking noise, while lower-frequency songs were (Halfwerk et al., 2011).

Despite this one study, there is still little direct evidence that spectrally-adjusted songs actually improve audibility in typical urban noise conditions. It is, for example, possible that birds may sing higher as a by-product of singing louder, without additional benefits (Verzijden et al., 2010; Nemeth et al., 2013). Another possibility is that spectral adjustment may come at the cost of reducing the perceived quality of the signal (functional compromise hypothesis, Slabbekoorn & Ripmeester, 2008; Gross, Pasinelli & Kunc, 2010; Halfwerk et al., 2011; Slabbekoorn, 2013; Read, Jones & Radford, 2014). Thus, in playback studies, receivers may be less motivated to respond to spectrally-adjusted songs as they may be perceived as low-quality, even though they may be easier to detect (Des Aunay et al., 2014; Luther & Magnotti, 2014; but see Halfwerk et al., 2011). Finally, as anthropogenic noise tends to correlate with urbanization, in some cases it may be possible that spectral adjustments are not responses to elevated noise levels per se, but to other urban factors, such as increased aggression or boldness and territory density (Ripmeester et al., 2010; Hamao, Watanabe & Mori, 2011).

Black-capped chickadees have a single song type (*fee-bee* whistled song), but individual males are capable of pitch-shifting their songs up and down in frequency during male-male interactions (Otter et al., 2002). Pitch-shifting is thus a tool black-capped chickadees can, and do, use to spectrally adjust their vocalizations to higher frequencies in noisy conditions (Proppe et al., 2012; LaZerte, Slabbekoorn & Otter, 2016) or to shift away, up or down, from narrow bands of masking noise (Goodwin & Podos, 2013). Shifting to higher frequencies in noisy conditions is presumably adaptive as transmission studies show that black-capped chickadee songs are masked by anthropogenic noise (LaZerte, Otter & Slabbekoorn, 2015). Furthermore, black-capped chickadees do not appear to have an innate tendency to respond more or less to low- versus high-frequency songs; Frequency-matching, instead of absolute frequency, is the important signal in male-male interactions (Mennill & Otter, 2007), while females do not appear to differentiate at all (Ratcliffe & Otter, 1996). This makes the black-capped chickadee an ideal candidate, compared to other species, for testing whether high-frequency songs are actually more audible than low-frequency songs in noisy conditions.

Here we tested whether black-capped chickadees responded differently to high- vs low-frequency songs in a playback experiment combining song and experimental noise. We experimentally controlled for song amplitude and habitat-related effects by standardizing playback amplitude and conducting studies across gradients of habitat urbanization. Furthermore, as black-capped chickadees normally sing both high and low songs, paired playbacks were created from a single individual, controlling for individual effects. Low-frequency songs should broadcast better in general under low-noise conditions than high-frequency songs—due both to frequency-dependent attenuation and propensity for scatter and reverberation (Wiley & Richards, 1982; Brown & Handford, 2000). Only under conditions of low-frequency noise would we expect higher audibility of high-frequency songs, and thus differential response by male chickadees. We played song stimuli embedded in traffic-like low-frequency noise, starting with quiet songs which gradually increased in amplitude relative to simulated background noise. We hypothesized that songs which are more audible in noise will be detected earlier at lower amplitudes and will therefore elicit quicker reactions. The literature suggests that black-capped chickadees do not differentiate between absolute song frequencies, but to confirm this we also examined relative aggression levels directed at the two song frequencies. As a previous study demonstrated that spectral plasticity in black-capped chickadees depends on familiarity with noise, we also controlled for local ambient noise in each trial (LaZerte, Slabbekoorn & Otter, 2016). Therefore, we asked two main questions: When stimuli are embedded in experimental noise and controlling for local ambient noise levels, do black-capped chickadees respond (1) more quickly or (2) more aggressively to high- vs low-frequency songs?

## Methods

### Site and timing

We performed playback trials in and around the cities of Prince George, Quesnel and Vancouver, British Columbia, Canada, between 5 April and 18 April 2012, and between 27 April and 3 May 2013. Trials were conducted across a variety of landscapes from highly urbanized to completely undisturbed rural, as well as across an amplitude gradient of ‘naturally’-occurring anthropogenic ambient noise. Twenty-four male black-capped chickadees were successfully exposed to matched-stimuli playback trials (dyads). Each focal male was presented with one trial containing low-frequency stimulus-songs and one containing high-frequency stimulus-songs embedded in background noise. Playback order (High/Low vs Low/High) alternated between focal males, and in total we exposed 13 males to the High/Low order of stimuli and 11 males to the Low/High order (total sample size of 24 males, and 48 playback trials). The playbacks for each male were presented in the morning between 0700 and 1200 and dyadic stimuli were separated by a median of 1.1 h (High/Low median 1.1 h, range 1.0–4.4 h; Low/High median 1.1 h, range 0.7–1.8 h). Neighbours were never tested on the same day. To avoid pseudoreplication of playback stimuli, we used 17 unique stimuli sets and played each set to a maximum of two focal males (once ordered High/Low, and once Low/High).

### Playback files

**Figure 1:**
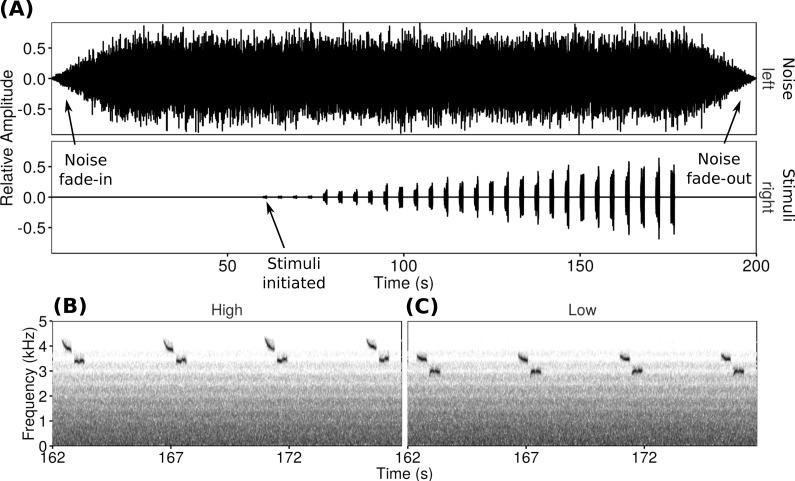
Playback trials consisted of a WAV file with one channel of noise and one channel of songs repeated at increasing amplitudes. The oscillogram (A) shows the increasing song amplitude as well as the noise fade-in and fade-out. Spectrograms show examples of the two types of songs used at the loudest amplitude (high-frequency (B) and low-frequency (C)).

All songs used in playbacks were unique and obtained from dawn chorus recordings of 11 male black-capped chickadees from Prince George, Kamloops, Kelowna and Vancouver in 2011 and 2012 (LaZerte, Slabbekoorn & Otter, 2016). All songs were from individuals unfamiliar to the focal male. Song frequencies were defined as the dominant frequency of the second ‘*bee*’-note (cf. Christie, Mennill & Ratcliffe, 2004; LaZerte, Slabbekoorn & Otter, 2016). Because black-capped chickadees routinely pitch-shift their songs, it was possible to get both low- and high-frequency songs from recordings of a single individual. Therefore, all stimuli dyads were created from low-frequency (2.99–3.21 kHz) and high-frequency (3.34–3.50 kHz) songs recorded from a single male black-capped chickadee (Fig. 1B). Within a playback dyad, frequency between high- and low-frequency songs differed by an average of 0.33 kHz (range 0.18–0.44 kHz). By pairing trials so that each focal male only heard songs from a single individual, we controlled for effects of individual dominance or variation in song consistency (Grava, Grava & Otter, 2013). Background sounds were removed from recordings and songs were normalized to a constant volume prior to use.

For each playback trial a WAV file with two channels was created. The left channel broadcast synthetic noise progressively filtered resulting in a frequency spectrum simulating traffic noise (cf. LaZerte, Slabbekoorn & Otter, 2016), while the right channel broadcast the stimulus songs with increasing amplitude as the trial progressed (Fig. 1). The noise started 60 s before the song stimuli, including 20 s of fade-in to full volume followed by 40 s of full volume noise to allow the focal males to acclimate. At the end of the trial, the experimental noise faded out over 20 s. The song channel consisted of four unique songs spaced approximately once every 4 s. This sequence was repeated (increasing in amplitude over each repeat) seven times over 2 min (rate of 14 songs/min to a total of 28 songs over two minutes). Over the course of a trial the signal-to-noise ratios of the songs in the right channel compared to the background noise in the left channel ranged from −16 dB at the start to −1 dB at the end of the playback.

The playback was broadcast from a Roland Mobile Cube amplifier (Roland Incorporation, USA; ‘Full range audio’ frequency response ∼100 Hz–20 kHz) connected to a Philips GoGear Raga MP3 player (Philips Ltd., Canada) at a volume so that background noise was ∼68 dB(Z) (63 dB(A)) at 5 m and the loudest song (without background noise) was ∼67 dB(Z) (64 dB(A)) at 5m. As the left and right channels correspond to the two side-by-side speakers in these amplifiers, noise and stimuli were broadcast from the same direction from the perspective of the focal male.

### Playback trials

To ensure the focal male was within range and responsive, we started all playback trials by priming with a series of recorded black-capped chickadee *chick-a-dee* calls (e.g., Grava, Grava & Otter, 2013). Priming consisted of 12 calls presented over 30 s; if the focal male responded and approached the playback speaker, we stopped the calls and initiated the playback sequence. If there was no response after 30 s of calls, we waited for 2 min and restarted priming calls. If males failed to respond to a second or a third sequence of priming, trials were aborted until at least the following day. Conversely, if males were detected prior to initiating priming calls, we still played at least two individual priming calls. In this manner, all males received at least two calls to ensure similar motivation and attention between trials. As there was at least a 1-minute delay between the last priming call and the first song stimuli broadcast, it was not possible to standardize focal male position. We therefore omitted focal males which were either too close (<5 m) and so could have easily perceived faint stimuli, or too far (>25 m) and so might not have heard the stimuli at all.

During the trial, a MKH70 Sennheiser microphone (Sennheiser Inc., Canada) was used to record dictated focal male movements and vocalizations onto a Marantz PMD671 Digital recorder (Marantz Canada, LLC; 22 bit and 44.1 kHz sampling frequency). Distances were measured by eye by the same observer (SEL) in all trials. Ropes marked at 5 m and 10 m distances were stretched away from the speaker in four directions and were used to aid distance estimates. A Bushnell Sport 850 laser rangefinder (Bushnell Outdoor Products Canada, Canada) was used during and/or after the trial to confirm perch heights.

‘Naturally’-occurring ambient noise levels were characterized for each site after each trial. Measurements were made with a Pulsar 30 sound pressure level meter (Pulsar Instruments plc., UK) and averaged across trials to obtain a measure of the general site noise levels experienced by the focal male. The general noise levels experienced by focal males at each site (local ambient noise) ranged from 56 to 71 (median 65) dB(Z) for sites where we played High/Low playback pairs and from 53 to 71 (median 64) dB(Z) where we played Low/High playback pairs. Previous studies have shown that urban habitat structure has little effect on transmission of chickadee song relative to signal interference by urban noise conditions (LaZerte, Otter & Slabbekoorn, 2015) and that it does not influence spectral adjustment in black-capped chickadees (LaZerte, Slabbekoorn & Otter, 2016). Thus, although we conducted our study across a variety of habitat types to ensure an even sampling, we did not evaluate effects of habitat urbanization.

### Focal male responses

During playback trials we tracked when vocalizations and movements were made, and the distance to the speaker each time the focal male moved. From these observations we defined three measures of focal male response to the playback: (1) Latency to first reaction (s), reflecting the time a focal male took to either start singing or fly more than 2 m towards the speaker; (2) a principal component index of aggression reflecting greater time spent close to the speaker (determined by approach speed and length of stay); and (3) a second principal component index of aggression reflecting greater time spent at intermediate distances and more singing by the focal male. These two measures of aggression are commonly used metrics among playback studies in black-capped chickadees (e.g., Shackleton, Ratcliffe & Weary, 1992; Otter et al., 2002; Grava, Grava & Otter, 2013).

We used base R (v3.3.2, R Core Team, 2016) to calculate the two indices of aggression through principal component analysis. Variables included were: time spent within various distance categories (s), latency to the closest approach to the speaker (s), the closest approach (m), and the total number of songs sung. We evaluated only principal component (PC) axes with greater total variance explained than the broken stick model, given the number of variables (Legendre & Legendre, 1998). We only interpreted contributions which were greater than 0.33 (Ho, 2006). High scores on the first principal component axis (PC1) reflected birds spending more time close to the speaker (<10 m), spending less time far from the speaker (>20 m), taking longer to get to the closest distance, but getting closer to the speaker overall (PC1: *Approach and stay close*; Table 1). High scores on the second principal component axis (PC2) reflected birds spending less time at intermediate distances (10–20 m), spending more time far from the speaker (>20 m), and singing less (Table 1). We multiplied PC2 loadings by −1 in order to create an index reflecting greater time spent at intermediate distances, less time spent farther away, and more songs sung (PC2: *Sing more*).

**Table 1:** Principal component analysis of black-capped chickadee responses to playback stimuli. Bold values reflect variables with contributions of greater than 0.33. Note that PC2 loadings were multiplied by −1 prior to use to reflect overall *increases* in songs sung and time spent at intermediate distances.

Parameter	PC1	PC2
Time <10 m	**0.54**	0.26
Time 10–20 m	−0.16	−**0.75**
Time >20 m	−**0.48**	**0.39**
Latency to min dist.	**0.38**	−0.12
Min distance	−**0.53**	−0.11
Total songs sung	0.19	−**0.44**
Total variance explained	**0.45**	0.25

### Statistical analysis

All analyses were performed with R statistical software (v3.3.2, R Core Team, 2016). We analyzed our three focal male responses (Latency, PC1 and PC2) with linear mixed models using focal male ID as a random factor to account for the repeated measures design (R package lme4 v1.1-12, Bates et al., 2016). Degrees of freedom were calculated using the Satterthwaite approximation (R package lmerTest v2.0-32, Kuznetsova, Brockhoff & Christensen, 2016). These degrees of freedom coupled with the *t*-statistic provided by the lme4 package were used to calculate corresponding *P*-values.

Preliminary analysis suggested that in addition to playback stimulus type (High vs Low), playback order (whether the high-frequency stimulus was presented first, High/Low, or the low-frequency stimulus was presented first, Low/High) was an important factor. We therefore categorized all playback trials by both stimulus frequency and playback order. This resulted in four categories: “High (High/Low)”, “Low (High/Low)”, “High (Low/High)”, “Low (Low/High)”. Differences between responses were then evaluated with custom independent contrasts which tested for a within-subject effect of stimulus frequency within each order of playbacks as well as for a between-subject overall effect of playback order (Table 2).

**Table 2:** Custom independent contrasts for testing response differences between categories. Specifically we tested for (1) an effect of stimulus frequency when high-frequency stimuli were presented first (High/Low), (2) an effect of stimulus frequency when low-frequency stimuli were presented first (Low/High), and (3) for an overall effect of playback order.

Contrast	High (High/Low)	Low (High/Low)	High (Low/High)	Low (Low/High)
(1) Low vs High for High/Low	1	−1	0	0
(2) Low vs High for Low/High	0	0	1	−1
(3) High/Low vs Low/High	−1/2	−1/2	1/2	1/2

Previous work has shown that black-capped chickadees can adjust their vocalizations in response to ambient noise (LaZerte, Slabbekoorn & Otter, 2016). We therefore also included local ambient noise as an explanatory variable (centred around the mean). Because audibility can also be affected by distance, we included the starting distance between the focal male and the speaker as a covariate. We confirmed that there was no multicollinearity (all Variance Inflation Factors <7, condition numbers all <30, Quinn & Keough, 2002) and that the assumptions of constant variance and normality of errors were satisfied. Marginal and conditional R^2^s (Nakagawa & Schielzeth, 2013) were calculated in R with the MuMIn package (v1.15.6, Bartoń, 2016). All figures were produced with the R package ggplot2 (v2.2.1, Wickham, 2009). Spectrograms and oscillograms were produced with the R package seewave (v2.0.2, Sueur, Aubin & Simonis, 2008) with a Hanning window length of 1,024.

### Ethics

All work was carried out with approval from University of Northern British Columbia Animal Care and Use Committee (protocol No. 2011-05).

## Results

**Figure 2:**
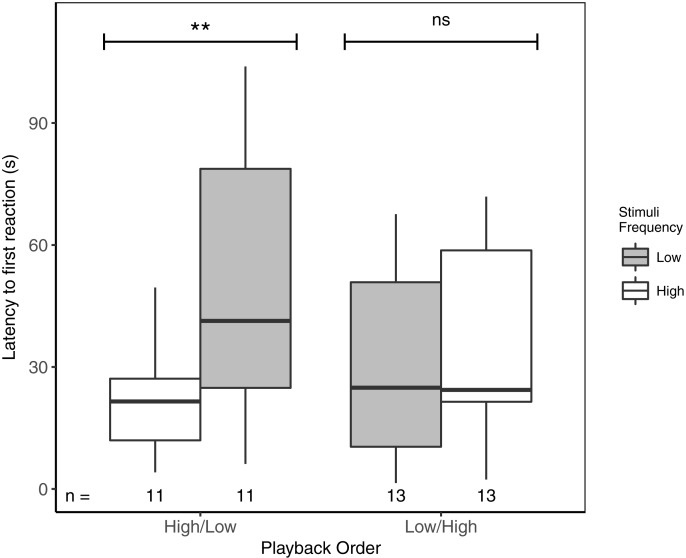
Male black-capped chickadees reacted more quickly to high- vs low-frequency stimuli, but only when high-frequency stimuli were presented first in paired trials. ** reflects a significant difference, ns, a non-significant difference. There was no overall difference between responses to High/Low vs Low/High trials. Boxplots reflect distribution of data. Boxes show 25th, 50th and 75th percentiles, ‘whiskers’ are to the minimum and maximum values within 1.5 × the inter-quartile range (IQR). Sample sizes for each trial are listed below each box. In total there were 48 trials for 24 males (2 trials each).

Latency to first response ranged from 1.5 to 103.9 s. Focal males responded significantly faster to high- vs low-frequency stimuli, but only when high-frequency stimuli were presented first (Table 3; Fig. 2). There was no overall effect of playback order, nor were there effects of ‘naturally’ varying ambient noise or starting distance (Table 3).

**Table 3:** Results of linear mixed models comparing responses to high- vs low-frequency stimuli in black-capped chickadees. The responses were Latency to first reaction, PC1 (*Approach and stay close*), and PC2 (*Sing more*), and they were modelled against three contrasts (Table 2), local ambient noise (dB(Z)) and starting distance (m). Slope Est ± CI 95% refers to slope parameter estimates ± the 95% confidence intervals. *df* represents degrees of freedom. Bold lines ending with an asterisk indicate significant effects (*P* < 0.05). In each analysis, parameters in the bottom two rows represent variance of random effects (male ID) and the residual, as well as marginal and conditional *R*^2^. Each analysis included responses from 24 focal males and 48 individual trials.

Analysis	Parameter	Slope Est. ± CI 95%	*df*	*t*	*P*
Latency to first response (s)	Intercept	**40.18** ± **21.00**	42	3.75	0.001*
	Low vs High (High/Low)	−**13.70** ± **9.77**	21	−2.75	0.012*
	Low vs High (Low/High)	3.51 ± 8.92	21	0.77	0.449
	Low/High vs High/Low	−2.26 ± 15.23	20	−0.29	0.774
	Local ambient noise (dB(Z))	0.81 ± 1.72	22	0.92	0.365
	Starting distance (m)	−0.46 ± 1.44	39	−0.63	0.531
	Random variance	90.51 (male ID)	536.97 (residual)		
	*R*^2^	0.17 (marginal)	0.29 (conditional)		
PC1 (*Approach and stay close*)	Intercept	**1.58** ± **1.32**	41	2.34	0.024*
	Low vs High (High/Low)	−0.20 ± 0.58	22	−0.68	0.503
	Low vs High (Low/High)	0.05 ± 0.53	22	0.19	0.853
	Low/High vs High/Low	0.02 ± 1.04	21	0.03	0.974
	Local ambient noise (dB(Z))	−0.07 ± 0.12	23	−1.18	0.250
	Starting distance (m)	**−0.12** ± **0.09**	37	−2.54	0.016*
	Random variance	0.70 (male ID)	1.92 (residual)		
	*R*^2^	0.13 (marginal)	0.36 (conditional)		
PC2 (*Sing more*)	Intercept	−0.07 ± 0.93	40	−0.15	0.878
	Low vs High (High/Low)	−0.01 ± 0.41	20	−0.04	0.967
	Low vs High (Low/High)	0.08 ± 0.37	20	0.42	0.679
	Low/High vs High/Low	**1.04** ± **0.74**	19	2.77	0.012*
	Local ambient noise (dB(Z))	0.04 ± 0.08	21	1.01	0.325
	Starting distance (m)	0.00 ± 0.06	35	0.07	0.946
	Random variance	0.38 (male ID)	0.92 (residual)		
	*R*^2^	0.19 (marginal)	0.43 (conditional)		

**Figure 3:**
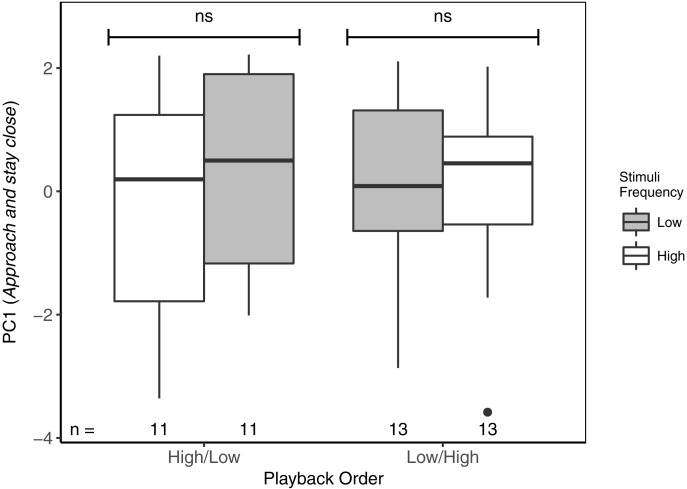
Focal males did not differentiate in PC1 (*Approach and stay close*) between high- and low-frequency stimuli regardless of playback order. ns reflects a non-significant difference. There was no overall difference between responses to High/Low vs Low/High trials. Boxplots reflect distribution of data. Boxes show 25th, 50th and 75th percentiles, ‘whiskers’ are to the minimum and maximum values within 1.5 × the inter-quartile range (IQR). Points are values outside of 1.5 × IQR. Sample sizes for each trial are listed below each box. In total there were 48 trials for 24 males (2 trials each).

With respect to PC1 (*Approach and stay close*), focal males did not differentiate between high- and low-frequency stimuli, regardless of playback order (Table 3; Fig. 3). Neither was there an overall effect of playback order, nor of local ambient noise. However, starting distance was negatively correlated with PC1, indicating that as starting distance decreased, PC1 increased (individuals approached and spent more time near the speaker).

**Figure 4:**
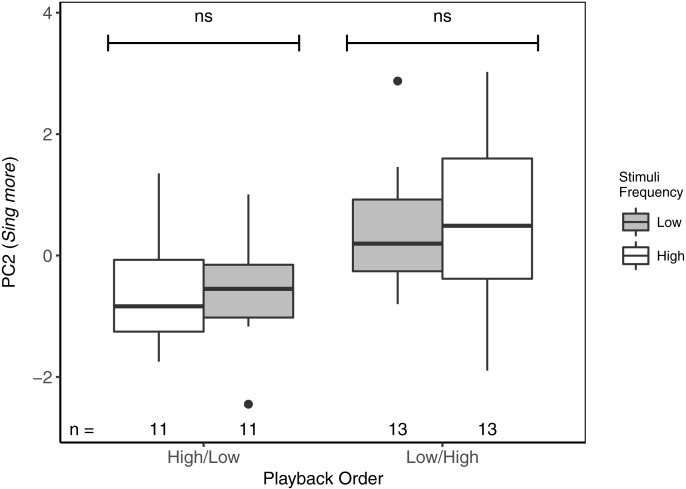
While there were no differences in PC2 (*Sing more*) between playbacks of high- and low-frequency stimuli, focal black-capped chickadees sang more in response to both stimuli when low-frequency stimuli were presented first. ns reflects a non-significant difference. Overall, males responded significantly more to Low/High than to High/Low trials. Boxplots reflect distribution of data. Boxes show 25th, 50th and 75th percentiles, ‘whiskers’ are to the minimum and maximum values within 1.5 × the inter-quartile range (IQR). Points are values outside of 1.5 × IQR. Sample sizes for each trial are listed below each box. In total there were 48 trials for 24 males (2 trials each).

For PC2 (*Sing more*), there were also no differences between responses to high- vs low-frequency stimuli, regardless of the playback order (Table 3; Fig. 4). There was, however, an overall effect of playback order, such that males sang more overall when low-frequency stimuli were presented first. There were no effects of local ambient noise nor of starting distance.

## Discussion

We found that black-capped chickadees responded faster to high- than to low-frequency songs when embedded in traffic-like low-frequency noise, but only when the playback order was High/Low. This suggests that in black-capped chickadees, high-frequency songs may be easier to detect in noisy conditions than low-frequency songs. We found no evidence that black-capped chickadees respond more or less aggressively to high- or low-frequency songs, and there is therefore no indication that chickadees discriminate between songs of high and low frequencies, once the song has been detected. Furthermore, we observed no effect of local ambient noise on relative response strength to high- vs low-frequency songs, suggesting that local noise conditions do not influence the responses of focal males.

### The role of frequency in signal detection

Differences in response latency may be the result of combined effects of masking-related differences in audibility and a carry-over effect of delayed responses to the second stimulus due to habituation. Playback order can influence how individuals react to the stimuli (see e.g., Richards, 1981; Naguib, 1999). In our study, slower responses to the low-frequency songs when the playback order was High/Low but no difference in response latency when the order was Low/High could be explained by the combined effects of detecting high-frequency songs more rapidly, but simultaneously responding more slowly to the second playback due to habituation. This would result in a large difference in responses between high- and low-frequency songs when high-frequency songs are presented first (both more audible and the first playback) but an equivalent response when low-frequency songs are presented first (less audible, but presented first). A two-factorial design, adding High/High and Low/Low playback combinations could be used to confirm this interpretation. Alternatively, allowing a longer time period between playback trials may reduce the effect of playback order, but with the side-effect of introducing variation in weather, temperature, or other potential confounds.

Focal males may have responded more quickly to high- than to low-frequency songs because high-frequency songs were more audible, but they also may have responded more quickly because high-frequency songs were perceived as being a greater threat (and worthy of early response, or responding when the perceived threat was farther away). However, in this study, stimuli within a paired playback (High vs Low) originated from the same recording of a single black-capped chickadee. As such, between stimuli within a pair there would be little to no acoustic variation due to the recording’s year, season, habitat, time of day, or individual motivation. The only feature which may have affected the perceived threat is song frequency.

Frequency-matching during intra-sexual singing bouts indicates a male black-capped chickadee’s willingness to escalate contests (Horn et al., 1992; Otter et al., 2002; Mennill & Ratcliffe, 2004; Fitzsimmons et al., 2008; Foote et al., 2008). If high-frequency songs were also used to communicate increased aggression, we would have expected to see significantly closer approaches to the speaker during playbacks of high- compared with low-frequency songs and we would have expected high-frequency songs to elicit more singing than low-frequency songs, neither of which occurred. Further, individuals could have responded more aggressively to high-frequency stimuli, simply because if detected earlier, these stimuli would have been perceived as a longer exposure to the ‘intruder’ than would low-frequency stimuli detected later. However, we did not observe any differences in our aggression measures, suggesting either that longer exposure to high-frequency stimuli was balanced by slightly more aggressive responses to low-frequency stimuli, or that any differences were minor enough not to be detected.

We did see an overall effect of playback order on PC2 (*Sing more*), suggesting that low-frequency songs might have some sort of alerting effect, resulting in more songs being sung in response to both high- and low-frequency songs, but only when low-frequency songs were presented first (carry-over effect, cf. Richards, 1981; Naguib, 1999). This would suggest low-frequency songs were perceived as somewhat more aggressive signals than high-frequency songs. There is also the possibility that individuals in the Low/High group may have been stronger responders in general, but as individuals were assigned treatments through a random block design, this is unlikely. Regardless, even though males in the Low/High group showed higher overall responses to playbacks, they still did not differentiate between high- and low-frequency stimuli. Taken together, these lines of evidence suggest that black-capped chickadees did not perceive high-frequency stimuli as a more aggressive signal than low-frequency stimuli, and thus cannot explain why high-frequency song stimuli elicited earlier responses.

Although high frequencies correlate with density and potential aggression in some species (Ripmeester et al., 2010; Hamao, Watanabe & Mori, 2011), to our knowledge, only one study has even tentatively suggested that absolute frequency itself may reflect aggression in black-capped chickadees (Hill & Lein, 1987); In that study, lower-frequency songs were suggested to be the more aggressive signal. Subsequent playback studies, however, suggest that it is frequency-matching rather than frequency itself which is more important (Mennill & Otter, 2007), and another study found that female black-capped chickadees did not appear to differentiate between high- and low-frequency songs (Ratcliffe & Otter, 1996). Therefore, there is little evidence to suggest that absolute differences in frequency between the stimuli would in themselves have motivated focal males to quicker responses.

As all of our trials included background noise, it is possible that this difference in latency could be related more to the general acoustic attributes of high- vs. low-frequency songs, rather than to how they transmit in noisy conditions. However, in typical low-noise, forested, black-capped chickadee habitat, low-frequency songs would be expected to have better transmission, as higher frequencies suffer from greater attenuation and greater degradation due to scatter from vegetation (Wiley & Richards, 1982; Brown & Handford, 2000). Thus, in the absence of noise we would have expected low-frequency songs to transmit better than high-frequency songs, suggesting that the faster response to high-frequency songs in noisy conditions that we observed in this study is likely due to masking release. Only in conditions of low-frequency noise would high-frequency songs be predicted to be more audible, and thus elicit quicker reactions, than low-frequency songs.

### No effect of local ambient noise

In this study we found no effect of local ambient noise on either detection of or discrimination between high- and low-frequency songs. Noise is additive on a logarithmic scale; thus in our study it is likely that the addition of experimental noise increased overall noise levels in quiet areas to something comparable to a noisy habitat, but that in a noisy habitat the broadcast would have resulted in a lower-perceived increase in ambient noise levels. Therefore, any potential effects of ambient noise would likely reflect long-term, habitat-related differences in how receivers perceive noise. In the current study, however, habitat-related differences in receiver perception do not seem to be a factor.

In contrast to our study, other studies have found evidence of discrimination between spectrally-adjusted and non-adjusted signals among individuals from different habitats, with different levels of background noise (great tits, Mockford & Marshall, 2009; European blackbirds, Ripmeester, Mulder & Slabbekoorn, 2010; northern cardinals, Luther & Magnotti, 2014; mountain chickadees Poecile gambeli, LaZerte, 2015). This may suggest that discrimination between signals can be influenced by habitat differences in how receivers perceive both signaller motivation and signal adjustment.

In our study, we may not have observed such differences because black-capped chickadees differ from many other species in that they produce both high- and low-frequency songs for use in pitch-matching interactions, where the absolute frequency does not appear to reflect individual quality (Mennill & Otter, 2007). In a previous study, we have shown that use of higher frequencies in male black-capped chickadees correlates with local ambient noise levels, and that territorial males adjust their frequency use to sudden, experimental increases in noise. However, individuals still used low-frequency songs and the expected upwards shift in frequency was dependent on experience with relatively high local noise levels (showing the apparent need for “learning to cope”; LaZerte, Slabbekoorn & Otter, 2016). Consequently, although high-frequency songs could vary in familiarity among male black-capped chickadees in different habitats, they are unlikely to ever represent a trade-off between signal audibility and signal content (functional compromise hypothesis; Slabbekoorn & Ripmeester, 2008; Slabbekoorn, 2013; Read, Jones & Radford, 2014).

### Conclusions

Playback order had an interactive effect on how quickly territorial males responded such that males initially exposed to high-frequency stimuli responded more quickly to high- than to low-frequency stimuli, but males initially exposed to low-frequency stimuli responded equally quickly to the two treatments. This suggests an interactive effect of masking release in high-frequency songs combined with habituation to the second stimulus; all other possible explanations for a difference in audibility would have predicted a quicker response to low- rather than high-frequency songs. Our work supports the findings of laboratory and field studies on great tits, which have shown easier detection of high- vs low-frequency songs in noisy urban conditions (Pohl et al., 2009; Pohl et al., 2012; Halfwerk et al., 2011). Future studies could help to clarify the role of playback order and add further solidity to our findings. Many studies have addressed the signaller’s perspective of the potential impact of anthropogenic noise and we therefore believe our study provides interesting new insights from the receiver’s perspective. We argue that comparative work on all aspects of communication, including descriptive and experimental data, continues to be important for a better understanding of how animals cope in a noisy world.

## Additional Information and Declarations

### Data Availability

The following information was supplied regarding data availability: Lazerte, Stefanie; Slabbekoorn, Hans; Otter, Ken (2017): Territorial black-capped chickadee males respond faster to high- than to low-frequency songs in experimentally elevated noise conditions. figshare.
https://doi.org/10.6084/m9.figshare.4246694.v1.

## References

[ref-1] Barber JR, Crooks KR, Fristrup KM (2010). The costs of chronic noise exposure for terrestrial organisms. Trends in Ecology & Evolution.

[ref-2] Bartoń K (2016). http://CRAN.R-project.org/package=MuMIn.

[ref-3] Bates D, Maechler M, Bolker B, Walker S (2016). http://CRAN.R-project.org/package=lme4.

[ref-4] Bermúdez-Cuamatzin E, Ríos-Chelén AA, Gil D, Garcia CM (2011). Experimental evidence for real-time song frequency shift in response to urban noise in a passerine bird. Biology Letters.

[ref-5] Brown TJ, Handford P (2000). Sound design for vocalizations: quality in the woods, consistency in the fields. The Condor.

[ref-6] Brumm H, Slabbekoorn H (2005). Acoustic communication in noise. Advances in the Study of Behavior.

[ref-7] Christie PJ, Mennill DJ, Ratcliffe LM (2004). Pitch shifts and song structure indicate male quality in the dawn chorus of black-capped chickadees. Behavioral Ecology and Sociobiology.

[ref-8] Des Aunay GH, Slabbekoorn H, Nagle L, Passas F, Nicolas P, Draganoiu TI (2014). Urban noise undermines female sexual preferences for low-frequency songs in domestic canaries. Animal Behaviour.

[ref-9] Fitzsimmons LP, Foote JR, Ratcliffe LM, Mennill DJ (2008). Frequency matching, overlapping and movement behaviour in diurnal countersinging interactions of black-capped chickadees. Animal Behaviour.

[ref-10] Foote JR, Fitzsimmons LP, Mennill DJ, Ratcliffe LM (2008). Male chickadees match neighbors interactively at dawn: support for the social dynamics hypothesis. Behavioral Ecology.

[ref-11] Francis CD (2015). Vocal traits and diet explain avian sensitivities to anthropogenic noise. Global Change Biology.

[ref-12] Goodwin SE, Podos J (2013). Shift of song frequencies in response to masking tones. Animal Behaviour.

[ref-13] Grava T, Grava A, Otter KA (2013). Habitat-induced changes in song consistency affect perception of social status in male chickadees. Behavioral Ecology and Sociobiology.

[ref-14] Gross K, Pasinelli G, Kunc HP (2010). Behavioral plasticity allows short-term adjustment to a novel environment. The American Naturalist.

[ref-15] Halfwerk W, Bot S, Buikx J, Velde M van der, Komdeur J, Ten Cate C, Slabbekoorn H (2011). Low-frequency songs lose their potency in noisy urban conditions. Proceedings of the National Academy of Sciences of the United States of America.

[ref-16] Hamao S, Watanabe M, Mori Y (2011). Urban noise and male density affect songs in the great tit *Parus major*. Ethology Ecology & Evolution.

[ref-17] Hill BG, Lein MR (1987). Function of frequency-shifted songs of black-capped chickadees. Condor.

[ref-18] Ho R (2006). Handbook of univariate and multivariate data analysis and interpretation with SPSS.

[ref-19] Horn AG, Leonard ML, Ratcliffe L, Shackleton SA, Weisman RG (1992). Frequency variation in songs of black-capped chickadees (*Parus atricapillus*). The Auk.

[ref-20] Hu Y, Cardoso GC (2009). Are bird species that vocalize at higher frequencies preadapted to inhabit noisy urban areas?. Behavioral Ecology.

[ref-21] Kleist NJ, Guralnick RP, Cruz A, Francis CD (2016). Anthropogenic noise weakens territorial response to intruder’s songs. Ecosphere.

[ref-22] Kuznetsova A, Brockhoff PB, Christensen RHB (2016). lmerTest: tests in Linear Mixed Effects Models. http://CRAN.R-project.org/package=lmerTest.

[ref-61] LaZerte SE (2015). Sounds of the city: the effects of urbanization and noise on mountain and black-capped chickadee communication. Doctoral Thesis.

[ref-23] LaZerte SE, Otter KA, Slabbekoorn H (2015). Relative effects of ambient noise and habitat openness on signal transfer for chickadee vocalizations in rural and urban green-spaces. Bioacoustics.

[ref-24] LaZerte SE, Otter KA, Slabbekoorn H (2017). Mountain chickadees adjust songs, calls and chorus composition with increasing ambient and experimental anthropogenic noise. Urban Ecosystems.

[ref-25] LaZerte SE, Slabbekoorn H, Otter KA (2016). Learning to cope: vocal adjustment to urban noise is correlated with prior experience in black-capped chickadees. Proceedings of the Royal Society B: Biological Sciences.

[ref-26] Legendre P, Legendre L (1998). Numerical ecology.

[ref-27] Lohr B, Wright TF, Dooling RJ (2003). Detection and discrimination of natural calls in masking noise by birds: estimating the active space of a signal. Animal Behaviour.

[ref-28] Luther D, Magnotti J (2014). Can animals detect differences in vocalizations adjusted for anthropogenic noise?. Animal Behaviour.

[ref-29] Luther DA, Phillips J, Derryberry EP (2016). Not so sexy in the city: urban birds adjust songs to noise but compromise vocal performance. Behavioral Ecology.

[ref-30] McMullen H, Schmidt R, Kunc HP (2014). Anthropogenic noise affects vocal interactions. Behavioural Processes.

[ref-31] Mennill DJ, Otter KA, Otter KA (2007). Status signalling and communication networks in chickadees: complex communication with a simple song. Ecology and behavior of chickadees and titmice.

[ref-32] Mennill DJ, Ratcliffe LM (2004). Overlapping and matching in the song contests of black-capped chickadees. Animal Behaviour.

[ref-33] Mockford EJ, Marshall RC (2009). Effects of urban noise on song and response behaviour in great tits. Proceedings of the Royal Society B: Biological Sciences.

[ref-34] Moiron M, González-Lagos C, Slabbekoorn H, Sol D (2015). Singing in the city: high song frequencies are no guarantee for urban success in birds. Behavioral Ecology.

[ref-35] Naguib M (1999). Effects of song overlapping and alternating on nocturnally singing nightingales. Animal Behaviour.

[ref-36] Nakagawa S, Schielzeth H (2013). A general and simple method for obtaining R^2^ from generalized linear mixed-effects models. Methods in Ecology and Evolution.

[ref-37] Nemeth E, Brumm H (2010). Birds and anthropogenic noise: are urban songs adaptive?. The American Naturalist.

[ref-38] Nemeth E, Pieretti N, Zollinger SA, Geberzahn N, Partecke J, Miranda AC, Brumm H (2013). Bird song and anthropogenic noise: vocal constraints may explain why birds sing higher-frequency songs in cities. Proceedings of the Royal Society B: Biological Sciences.

[ref-39] Otter KA, Ratcliffe L, Njegovan M, Fotheringham J (2002). Importance of frequency and temporal song matching in black-capped chickadees: evidence from interactive playback. Ethology.

[ref-40] Pohl NU, Leadbeater E, Slabbekoorn H, Klump GM, Langemann U (2012). Great tits in urban noise benefit from high frequencies in song detection and discrimination. Animal Behaviour.

[ref-41] Pohl NU, Slabbekoorn H, Klump GM, Langemann U (2009). Effects of signal features and environmental noise on signal detection in the great tit, *Parus major*. Animal Behaviour.

[ref-42] Proppe DS, Avey MT, Hoeschele M, Moscicki MK, Farrell T, St Clair CC, Sturdy CB (2012). Black-capped chickadees *Poecile atricapillus* sing at higher pitches with elevated anthropogenic noise, but not with decreasing canopy cover. Journal of Avian Biology.

[ref-43] Proppe DS, Sturdy CB, St. Clair CC (2013). Anthropogenic noise decreases urban songbird diversity and may contribute to homogenization. Global Change Biology.

[ref-44] Quinn GGP, Keough MJ (2002). Experimental design and data analysis for biologists.

[ref-45] Rabin LA, Greene CM (2002). Changes to acoustic communication systems in human-altered environments. Journal of Comparative Psychology.

[ref-46] R Core Team (2016). R: a language and environment for statistical computing.

[ref-47] Ratcliffe L, Otter K, Kroodsma DE, Miller EH (1996). Sex differences in song recognition. Ecology and evolution of acoustic communication in birds.

[ref-48] Read J, Jones G, Radford AN (2014). Fitness costs as well as benefits are important when considering responses to anthropogenic noise. Behavioral Ecology.

[ref-49] Richards DG (1981). Alerting and message components in songs of rufous-sided towhees. Behaviour.

[ref-50] Ripmeester EAP, Kok JS, Van Rijssel JC, Slabbekoorn H (2010). Habitat-related birdsong divergence: a multi-level study on the influence of territory density and ambient noise in European blackbirds. Behavioral Ecology and Sociobiology.

[ref-51] Ripmeester EAP, Mulder M, Slabbekoorn H (2010). Habitat-dependent acoustic divergence affects playback response in urban and forest populations of the European blackbird. Behavioral Ecology.

[ref-52] Shackleton S, Ratcliffe L, Weary D (1992). Relative frequency parameters and song recognition in black-capped chickadees. The Condor.

[ref-53] Slabbekoorn H (2013). Songs of the city: noise-dependent spectral plasticity in the acoustic phenotype of urban birds. Animal Behaviour.

[ref-54] Slabbekoorn H, Peet M (2003). Birds sing at a higher pitch in urban noise. Nature.

[ref-55] Slabbekoorn H, Ripmeester EAP (2008). Birdsong and anthropogenic noise: implications and applications for conservation. Molecular Ecology.

[ref-56] Sueur J, Aubin T, Simonis C (2008). seewave: a free modular tool for sound analysis and synthesis. Bioacoustics.

[ref-57] Verzijden MN, Ripmeester EAP, Ohms VR, Snelderwaard P, Slabbekoorn H (2010). Immediate spectral flexibility in singing chiffchaffs during experimental exposure to highway noise. The Journal of Experimental Biology.

[ref-58] Wickham H (2009). ggplot2: elegant graphics for data analysis.

[ref-59] Wiley RH, Richards DG, Kroodsma DE, Miller EH (1982). Adaptations for acoustic communication in birds: sound transmission and signal detection. Acoustic communication in birds: production, perception, and design features of sound.

[ref-60] Wood WE, Yezerinac SM (2006). Song sparrow (*Melospiza melodia*) song varies with urban noise. The Auk.

